# Which are the best Chinese herbal injections combined with XELOX regimen for gastric cancer? A PRISMA-compliant network meta-analysis

**DOI:** 10.1097/MD.0000000000010127

**Published:** 2018-03-23

**Authors:** Dan Zhang, Jiarui Wu, Kaihuan Wang, Xiaojiao Duan, Shi Liu, Bing Zhang

**Affiliations:** Department of Clinical Chinese Pharmacy, School of Chinese Materia Medica, Beijing University of Chinese Medicine, Beijing, China.

**Keywords:** Chinese herbal injections, gastric cancer, network meta-analysis, XELOX regimen

## Abstract

**Background:**

The optimal Chinese herbal injections (CHIs) combined with XELOX regimen for patients with gastric cancer remains elusive. The aim of our network meta-analysis (NMA) is to explore the best options among different CHIs for gastric cancer.

**Methods:**

PubMed, Embase, the Cochrane Library, the China National Knowledge Infrastructure Database (CNKI), Wan-fang Database, Cqvip Database (VIP), China Biology Medicine disc (CBMdisc) were searched to identify RCTs which focused on CHIs against gastric cancer. The quality assessment of included randomized controlled trials (RCTs) was conducted by the Cochrane risk of bias tool. Standard pair-wise and Bayesian NMAs were performed to compare the efficacy and safety of different CHIs combined with the XELOX regimen via Stata 13.0 and WinBUGS1.4 software.

**Results:**

A total of 2316 records were searched, the network of evidence included 26 eligible RCTs involving 13 types of CHIs and 2154 patients. The results suggested that Shenqifuzheng+ XELOX, Huachansu+ XELOX, Kangai+ XELOX, Javanica oil emulsion+ XELOX, Aidi injection+ XELOX might be the optimal treatment for gastric cancer in improving the performance status than using XELOX regimen single, with odds ratios (OR) and 95% confidence intervals (CIs) of 2.74 (1.24, 6.17), 8.27 (1.74, 42.43), 4.28 (1.80, 10.48), 5.14 (1.87, 16.28), 0.20 (0.090, 0.44). At the aspects of ADRs (adverse reactions), Compound Kushen+ XELOX, Lentinan+ XELOX, Xiaoaiping injection+ XELOX could obviously relieve leukopenia than only receiving XELOX regimen, and their ORs and 95% CIs were 5.62 (1.41, 36.24), 8.16 (2.25, 29.43), 5.69 (1.85, 15.77). Furthermore, Disodium cantharidinate and vitamin B6+ XELOX, Shenqifuzheng+ XELOX, Kangai+ XELOX, Lentinan+ XELOX could obviously relieve the nausea and vomiting than receiving the XELOX regimen alone, with ORs and 95% CIs of 5.29 (1.30, 23.96), 2.50 (1.16, 5.26), 2.42 (1.06, 5.63), 9.04 (3.24, 26.73). Nevertheless, CHIs combined with XELOX regimen did not confer higher better clinical effectiveness rate over receiving XELOX regimen alone, with nonstatistically significant between-group differences.

**Conclusions:**

As the available evidence suggested that CHIs combined with XELOX regimen could provide treatment benefits for patients with gastric cancer. Among 13 types of CHIs, Javanica oil emulsion and Compound Kushen injection is the optimal treatment in improving the clinical effectiveness rate and performance status, and Lentinan injection was superior in relieving ADRs.

## 1 Introduction

Gastric cancer is one of the commonly malignant tumors, remains the second leading cause of cancer death worldwide.^[1,2]^ Especially in Asia, gastric cancer remains a significant public health and an economic burden.^[3]^ The characteristics of gastric cancer include high morbidity and mortality,^[4]^ low surgical resection rate and 5-year overall survival rate.^[5,6]^ And its high mortality is closely associated with tumor invasion and metastasis.^[5]^ Still, except for surgery, chemotherapy and radiotherapy play important roles for treating gastric cancer.^[6]^ Capecitabine (Cap) in combination with oxaliplatin (L-OHP), namely the XELOX or CapeOX regimens, is considered one of the standard chemotherapy regimens for gastric cancer.^[7,8]^ Unfortunately, most cases receiving chemotherapeutic drugs are suffering treatment-related side-effects, drug resistance, and untoward complications.^[9]^ Therefore, patients with cancers are often unable to withstand the toxicities which may lead to a serious decline in the quality of life.^[10]^ As complementary and alternative medicine, Chinese herbal medicine is a popular treatment for gastric cancer nowadays owing to its functions of increasing efficacy and decreasing toxicity.^[11]^ Theoretically speaking, gastric cancer belongs to the category of “stomachache,” “dysphagia,” and “nausea” in Traditional Chinese Medicine (TCM).^[12]^ Recently, clinical practices have indicated that TCM play an increasingly important role in cancer therapy, because it can definitely improve the effects of chemotherapy and alleviate chemotherapy-induced ADRs.^[13–15]^ Moreover, the combinations of different CHIs with chemotherapy treatments have been proposed and applied in China.^[16,17]^ And the inherent advantages of CHIs are namely enhancing pharmacokinetic profile and intratumorous bioavailability compared with the TCM decoction.^[18,19]^

However, in spite of pharmacological and clinical research to explore the efficacy and safety of CHIs over the last decades, the optimal CHIs plus XELOX regimen treatment strategy for patients with gastric cancer remains unclear. To our knowledge, a NMA concerning the comparative efficacy and safety and of different CHIs plus XELOX regimen has not been previously accomplished. Hence, the aim of present study is to perform a NMA on this topic. As a new statistical method which is applied frequently in evidence-based medicine, NMA is developed from the conventional meta-analysis, and it possesses the advantages of simultaneous evaluating multiple interventions via Bayesian statistics. Furthermore, through collecting the relevant clinical trials, NMA can offer valuable evidences for clinical decision-making and recognize the superior options between different interventions which share a common network or chain.^[20–23]^ Given above, we conducted a Bayesian NMA of all relevant RCTs to identify the optimal CHIs plus XELOX regimen for patients with gastric cancer.

## 2 Methods

The current network meta-analysis was reported according to the PRISMA guidelines.

### 2.1 Database and search strategies

RCTs involving CHIs combined with XELOX regimen against gastric cancer were retrieved by searching the following databases from January 1979 to December 15, 2016: PubMed, the Cochrane library, Embase, CNKI, VIP, CBMdisc, and Wan-fang Database, without restrictions on language, date or type of publication. The search terms included 3 parts: gastric cancer, CHIs, and RCTs; the searching strategy adopted a combination of subject headings (MeSH) and free-text terms. In the Chinese databases, the search terms in CNKI about gastric cancer were “Stomach neoplasms, Gastric Neoplasms, Stomach Cancer,” with a full-text search for “random”; in English databases, the search terms for gastric cancer were “Stomach Neoplasm, Stomach Neoplasm, Gastric Neoplasms, Gastric Neoplasm, Stomach Cancer∗, Stomach Tumor∗, Gastric Cancer∗, Gastric Tumor∗, Gastric Carcinoma, Stomach Carcinoma.” The detailed search terms for each CHIs and specific retrieval strategies were summarized in Attachment 1. The reference lists of all retrieved articles were also scanned manually to identify any relevant trails. Parallel literature screenings were carried out by 2 reviewers independently. And we invited the specialists in information retrieval to make suggestions and amend our searching strategy. In the light of different electronic databases, we appropriately adjusted our search terms and search strategy to avoid suffering the problem of mismatching.

Since this study was a network meta-analysis of published RCTs and only involving previously published data, the ethical approval or informed consent was not required.

### 2.2 Eligibility criteria

All authors took participate in the establishment and incorporation of eligibility criteria for this NMA. Population: Our study participants were pathological or cytological diagnosed as gastric cancer. There was no restriction on gender, race, or nationality for the included patients. Interventions and Comparators: The CHIs group was treated by CHIs combined with XELOX regimen, and the XELOX group solely receiving XELOX regimen. The chemotherapeutic drugs of XELOX regimen were Cap and L-OHP. Outcomes: The outcomes for efficacy were the clinical effectiveness rate and performance status, and the safety outcomes were the ADRs involving leucopenia, nausea, and vomiting. The clinical effectiveness rate = [number of complete response patients + number of partial response patients]/total number of patients × 100%.^[24]^ Performance status was evaluated by the Karnofsky performance score (KPS): KPSs that increased more than10 points after treatment were considered to improve performance status. In terms of ADRs, it was calculated as: the incidence of ADRs = (number of patients occurred ADRs)/total number of patients × 100%.^[25]^ Study designs: Only RCTs were included in this NMA; nonrandomized controlled trials, cohort or case–control reports, editorials, letters, reviews, pharmacological or chemical experiments, and repeatedly published studies were excluded.

### 2.3 Data extraction and quality assessment

Two researchers (DZ and SL) extracted the following information from the included RCTs independently, and other 2 researchers (KW and XD) checked the standardized data extraction form: RCTs characteristics: title, the first authors’ names, publication date, and literature sources; and information about quality assessment. Baseline characteristics of patient: size, age, gender, KPSs before treatment, tumor types, and tumor stages and so forth. Intervention: the names, dosages, and treatment cycles of CHIs. Outcomes: the measured data about clinical effectiveness rate, performance status, and ADRs.

The 2 researchers (XD and KW) conducted the quality assessment of included RCTs by the Cochrane risk of bias tool (Cochrane Handbook, version 5.1.0.) independently. If there was disagreement occurred, discussion or further inquiry to a third researcher (XZ). The quality assessment items of Cochrane risk of bias tool included randomization, blinding, concealment of allocation, drop-outs, outcome reporting, other risk of bias.^[26]^ If there was disagreement between reviewers, a third researchers (DZ) would be available.

### 2.4 Statistical analysis

Firstly, the NMA were performed with Bayesian inference (WinBUGS 1.4.3, MRC Biostatistics Unit at Cambridge, United Kingdom). The dichotomous data were analyzed with a Bayesian random effects model with a binomial likelihood to calculate OR with 95% CI values between different treatment groups considering the clinical diversity among included RCTs.^[27,28]^ Based on the joint posterior distribution of all parameters, Bayesian inference in WinBUGS program calculated the posterior distributions of the interrogated nodes within the framework of the likelihood function via Markov Chain Monte Carlo (MCMC) simulation. We obtained 200,000 simulations, discarded the first 10,000 as burn-in and used the remainder iterations for inference.^[29]^ Secondly, we chose the Stata 13.0 software (Stata Corporation, College Station, TX) as graphical tools to present the results of statistical analyses in our NMA. For example, the network plot could illustrate the connection of the directly comparing different interventions from head-to-head trails, its nodes represented the interventions being compared and edges were represented as different intervention. And the width of each line is proportional to the number of trails; the size of each node was weighted according to the number of participants receiving the intervention.^[30,31]^ Meanwhile, the plots of the surface under the cumulative ranking probabilities (SUCRA) values were presented to rank the all competing treatments, with higher SUCRA scores reflecting higher associated efficacy and a lower rate of ADRs.^[32,33]^ Besides, clustering analysis was performed taken into account recommending an intervention for different outcomes simultaneously to identify the optimal CHIs.^[34,35]^ Furthermore, publication bias was assessed by funnel plots symmetry and using the Egger test and Begg test, with *P* < .05 suggesting obvious publication bias.^[36]^

## 3 Results

### 3.1 Literature search and the characteristics of included RCTs

**Figure 1:**
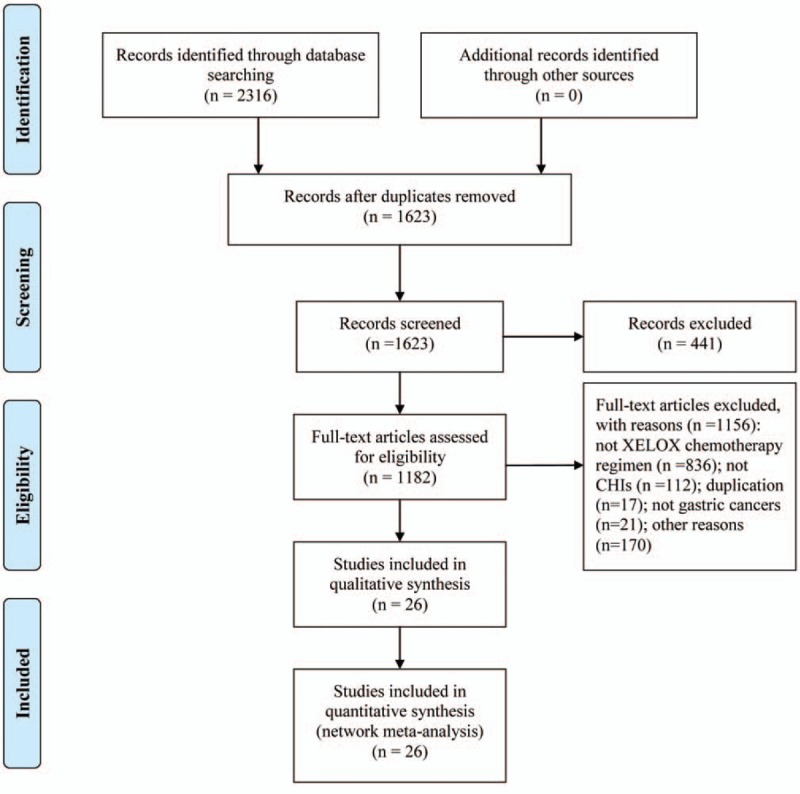
Flow chart of the search for eligible studies.

Following the process of PRISMA flow diagram in Fig. 1, a total of 2316 citations were indentified for potential inclusion in the present NMA electronic or manual searches. Finally, a total of 26 RCTs that evaluated CHIs combined with XELOX regimen against gastric cancer were included.^[37–62]^ Thirteen types of CHIs were identified, including Aidi, Shenmai, Xiaoaiping, Javanica oil emulsion, Kangai, Disodium cantharidinate and vitamin B6, Elemene, Lentinan, Astragalus polysaccharide, Huachansu, Compound Kushen, Shenqifuzheng, Shenfu injections.

**Figure 2:**
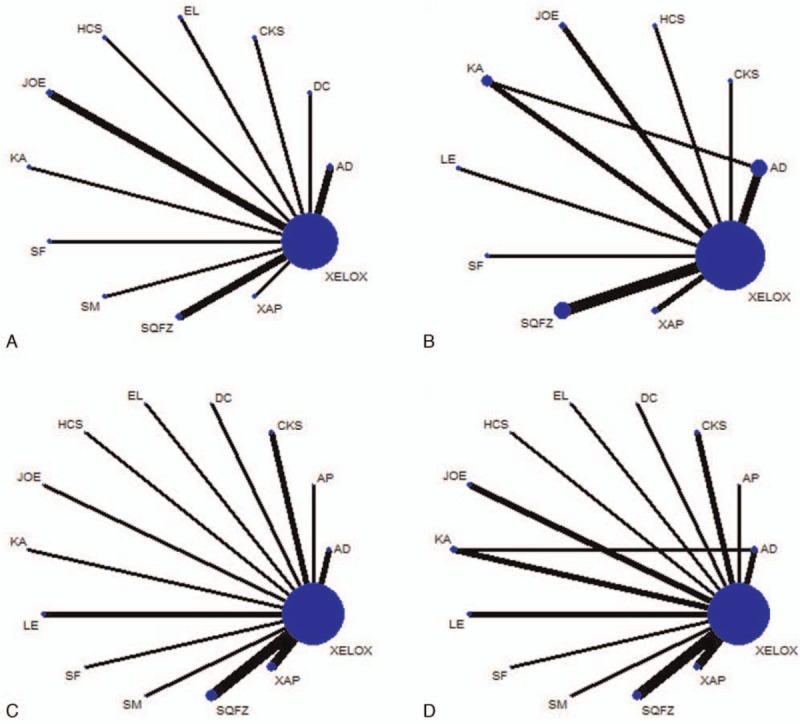
Network graph for 4 outcomes in this network meta-analysis. Note: (A) The clinical effectiveness rate; (B) performance status; (C) leucopenia; (D) nausea and vomiting. Node sizes indicate total sample sizes for treatments. Line thicknesses correspond to the number of trials used for comparisons.

The 26 RCTs included 13 types of CHIs and 2154 patients, among which 1150 patients were in CHIs group and 1004 were in XELOX groups.^[37–62]^ All of the included RCTs reported patient numbers and ages, while 21 (95.45%), 10 (45.45%), 9 (40.91%), and 11 (50.00%) trials reported the patients’ gender, tumor stages, expected survival time, and KPS before treatment, respectively. The baseline characteristics of each trial are provided in Table 1. And the network graph of 4 outcomes that compared different treatment groups is presented in Fig. 2.

**Table 1:**
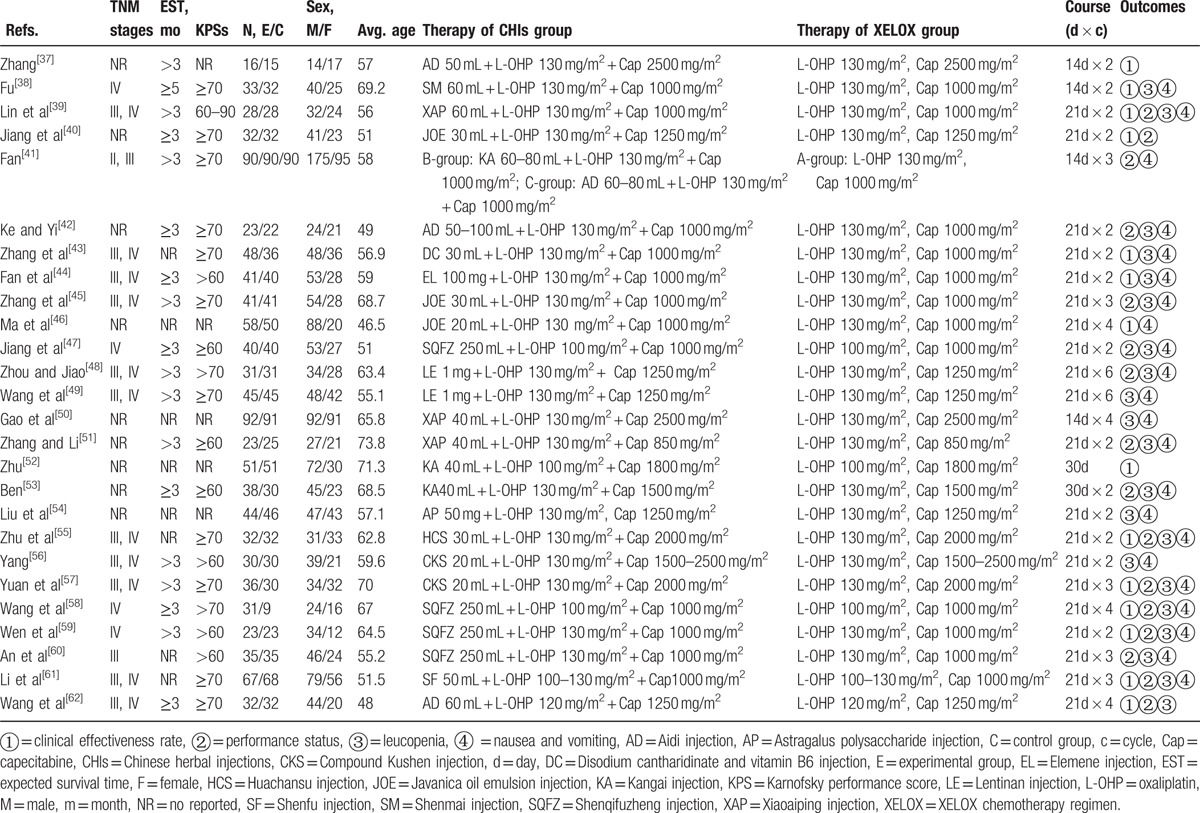
The basic characteristics of the included studies.

### 3.2 Quality assessment

**Figure 3:**
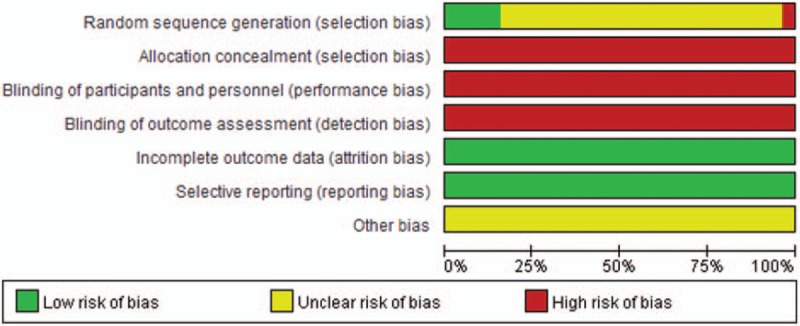
Risk of bias graph.

The results of quality assessment for included RCTs are shown in Fig. 3. Although all of the included RCTs mentioned randomization, 4 RCTs (15.38%) adopted the random number tables; 1 RCT (3.85%) applied the method of hospitalized time difference. Nevertheless, all of the included RCTs did not mention allocation concealment and blinding method. All of included RCTs did not select outcome reporting or have incomplete outcome data. And the included RCTs did not offer details about other bias. In addition, although the RCTs described the inclusion and exclusion criteria, they did not mention the sample size estimation and funding. Five among them (19.23%) reported the information about follow-up, survival rate or survival time.^[39,40,44,50,62]^ With regards to ADRs, 23 RCTs (88.46%) described the ADRs that was related to chemotherapeutic drugs. And 13 RCTs (50.00%) reported the details about medical ethics.

### 3.3 Outcomes

#### 3.3.1 The clinical effectiveness rate

**Figure 4:**
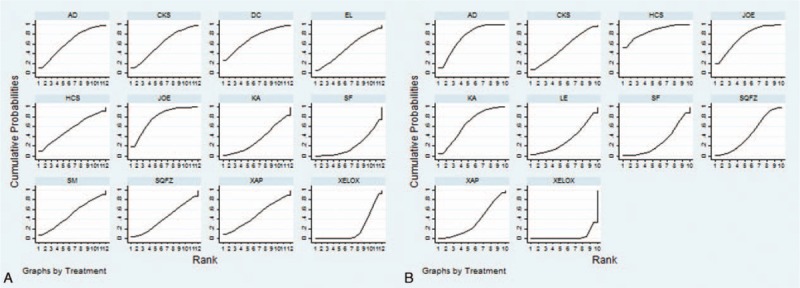
Rank of different treatment groups for efficacy outcomes. Note: (A) The clinical effectiveness rate; (B) performance status.

Fourteen RCTs with 11 types of CHIs reported the clinical effectiveness rate. The results for different CHIs groups in terms of the clinical effectiveness rate listed in Table 2. We detected nonsignificant difference between the CHIs groups (Aidi, Shenmai, Xiaoaiping, Javanica oil emulsion, Kangai, Disodium cantharidinate and vitamin B6, Elemene, Huachansu, Compound Kushen, Shenqifuzheng, and Shenfu injection) and XELOX group on patients with gastric cancer. Similarly, no obvious difference was observed among different CHIs groups. As illustrated in Fig. 4A, Javanica oil emulsion injection seemed to be the favorable option with regards to the clinical effectiveness rate, with SUCRA value was 72.51%. And other types of CHIs were ranked as follows: Compound Kushen (67.57%), Disodium cantharidinate and vitamin B6 (59.98%), Aidi (58.07%), Huachansu (56.28%), Elemene (51.74%), Shenmai (51.36%), Xiaoaiping (45.18%), Shenqifuzheng (44.17%), Kangai (39.7%), Shenfu (27.04%).

**Table 2:**
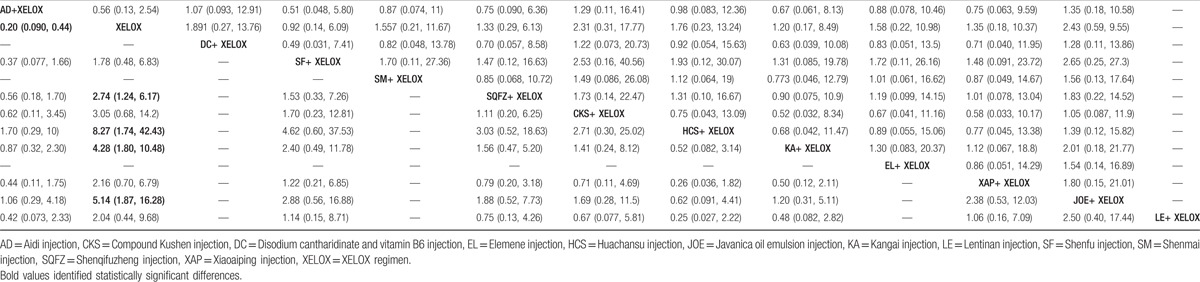
Results (ORs, 95% CIs) of the network meta-analysis of the clinical effectiveness rate (upper right quarter) and performance status (lower left quarter).

#### 3.3.2 Performance status

A total of 16 RCTs with 9 types of CHIs provided the information of performance status. The results indicated that among CHIs groups, Shenqifuzheng+ XELOX, Huachansu+ XELOX, Kangai+ XELOX, Javanica oil emulsion+ XELOX, Aidi injection+ XELOX were superior to using XELOX regimen single in improving performance status; these between-group differences were statistically significant, with ORs and 95% CIs of 2.74 (1.24, 6.17), 8.27 (1.74, 42.43), 4.28 (1.80, 10.48), 5.14 (1.87, 16.28), 0.20 (0.090, 0.44) (Table 2). According to the SUCRA values for each CHIs group that presented in Fig. 4B, Huachansu injection (85.88%) yielded significantly higher probability of improving the performance status than other CHIs, and the following rankings of CHIs were namely Javanica oil emulsion (74.17%), Aidi (73.47%), Kangai (66.45%), Compound Kushen (50.79%), Shenqifuzheng (45.07%), Xiaoaiping (35.68%), Lentinan (34.84%), Shenfu (28.35%).

#### 3.3.3 ADRs

##### 3.3.3.1 Leukopenia

**Figure 5:**
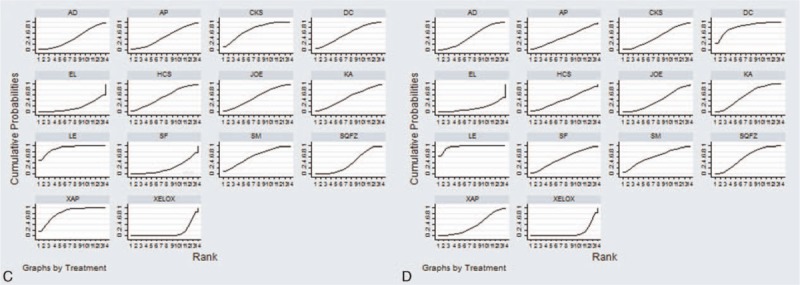
Rank of different treatment groups for ADRs. Note: (C) Leucopenia; (D) nausea and vomiting.

A total of 21 RCTs with 13 types of CHIs focused on the leukopenia. Table 3 offered the results of indirect comparisons concerning leucopenia, the results demonstrated that Compound Kushen+ XELOX, Lentinan+ XELOX, Xiaoaiping injection+ XELOX could obviously relieve leukopenia than XELOX group which only received XELOX regimen; and statistically significant differences were detected between these groups, with ORs and 95% CIs of 5.62 (1.41, 36.24), 8.16 (2.25, 29.43), 5.69 (1.85, 15.77). Based on the SUCRA values for leukopenia (Fig. 5C), Lentinan injection (87.84%) was associated with being a suitable treatment option for patients with gastric cancer in relieving leucopenia. And other CHIs were ranked as follows: Xiaoaiping (79.47%), Compound Kushen (77.82%), Shenmai (62.08%), Disodium cantharidinate and vitamin B6 (55.76%), Kangai (51.31%), Javanica oil emulsion (50.96%), Huachansu (50.68%), Astragalus polysaccharide (45.62%), Aidi (41.61%), Shenqifuzheng (40.12%), Shenfu (22.85%), Elemene (19.60%).

**Table 3:**
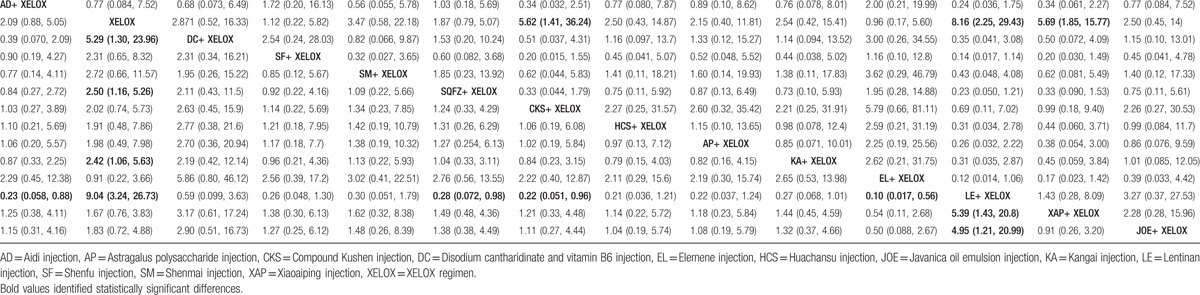
Results (ORs, 95% CIs) of the network meta-analysis of the leukopenia (upper right quarter), nausea and vomiting (lower left quarter).

##### 3.3.3.2 Nausea and vomiting

A total of 22 RCTs with 13 types of CHIs described the data of nausea and vomiting. The results of NMA showed that among CHIs groups, Disodium cantharidinate and vitamin B6+ XELOX, Shenqifuzheng+ XELOX, Kangai+ XELOX, Lentinan+ XELOX could achieve better effects on relieving the nausea and vomiting compared to XELOX group; the significant differences were identified among these groups, with ORs and 95% CIs of 5.29 (1.30, 23.96), 2.50 (1.16, 5.26), 2.42 (1.06, 5.63), 9.04 (3.24, 26.73). Furthermore, Lentinan+ XELOX was more effective in relieving the nausea and vomiting than Aidi+ XELOX, Shenqifuzheng+ XELOX, Compound Kushen+ XELOX, Elemene+ XELOX, Xiaoaiping+ XELOX, Javanica oil emulsion+ XELOX; these between-group differences were also statistically significant, with ORs and 95% CIs of 0.23 (0.058, 0.88), 0.28 (0.072, 0.98), 0.22 (0.051, 0.96), 0.10 (0.017, 0.56), 5.39 (1.43, 20.8), 4.95 (1.21, 20.99) (Table 3). Based on the above safety findings (Fig. 5D), Lentinan injection (95.47%) seemed to be the most tolerable therapy as it possessed the highest probabilities to relieve nausea and vomiting, and other CHIs were ranked as follows: Lentinan (95.47%), Disodium cantharidinate and vitamin B6 (82.60%), Shenmai (59.90%), Shenqifuzheng (58.71%), Kangai (56.98%), Shenfu (53.48%), Aidi (48.91%), Compound Kushen (47.31%), Astragalus polysaccharide (46.16%), Huachansu (44.67%), Javanica oil emulsion (42.07%), Xiaoaiping (37.33%), Elemene (15.87%). And the SUCRA values of each treatment groups with regards to 4 outcomes are summarized in Table 4.

**Table 4:**
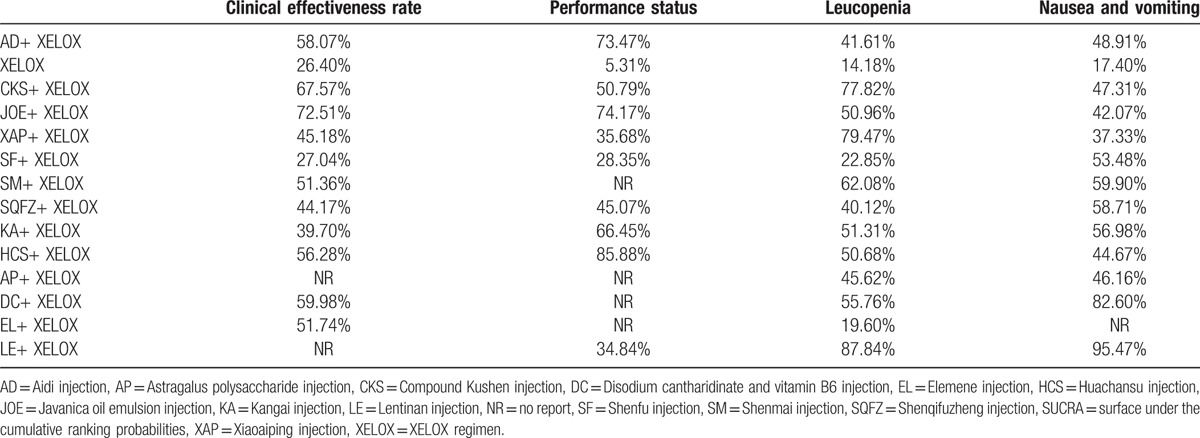
SUCRA values of 13 CHIs groups and XELOX group for outcomes.

#### 3.3.4 Cluster analysis

**Figure 6:**
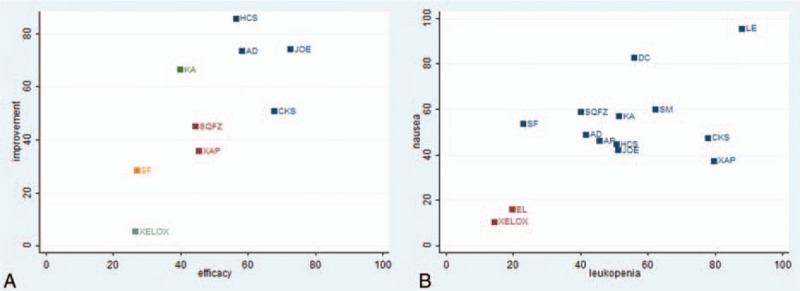
Cluster analysis plots of 4 outcomes. Note: (A) The clinical effectiveness rate and performance status (X-axis: the clinical effectiveness rate; Y-axis: performance status); (B) ADRs (X-axis: leukopenia; Y-axis: nausea and vomiting.).

Firstly, a cluster analysis was preformed for 8 types of CHIs that reported the outcomes of clinical effectiveness rate and performance status simultaneously. The cluster analysis plots were presented on the basis of SUCRA values, treatment groups with the same color belonged to the same clusters, and treatments that located in the upper right corner were related to the superior treatment benefits for improving the effectiveness rate and performance status. The results of the cluster analysis suggested that on the basis of XELOX regimen, Javanica oil emulsion, Huachansu, Aidi, Compound Kushen injections were associated with a better effectiveness rate and performance status. By contrary, receiving XELOX regimen single was the worst option in improving primary outcomes among these interventions (Fig. 6E). Secondly, a cluster analysis was conducted for 13 types of CHIs that reported both nausea and vomiting, and leukopenia. As shown in Fig. 6F, Lentinan injection was the most beneficial CHIs for alleviating ADRs in combination with XELOX regimen for patients with gastric cancer. However, using XELOX regimen single was the worst option in relieving ADRs.

#### 3.3.5 Publication bias

**Figure 7:**
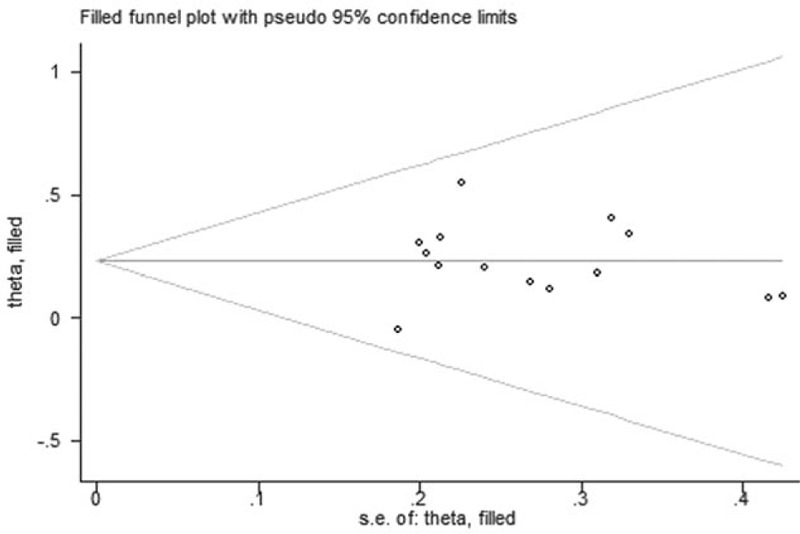
Funnel plot of performance status.

Publication bias and small-study effects were tested by funnel plots in terms of the clinical effectiveness rate (Fig. 7). The results of Egger test (*t* = 0.00, *P* = .99>.05) and Begg test (*z* = 0.66, *P* = .511>.05) showed no evidence of obvious publication bias among the included RCTs.

## 4 Discussion

The current NMA evaluated the clinical effectiveness rate, performance status, and ADRs for the combination of CHIs and XELOX regimen against gastric cancer. The results indicated that Shenqifuzheng+ XELOX, Huachansu+ XELOX, Kangai+ XELOX, Javanica oil emulsion+ XELOX, Aidi injection+ XELOX could achieve a significant improvement for performance status than using XELOX regimen single. As for ADRs, Compound Kushen+ XELOX, Lentinan+ XELOX, Xiaoaiping injection+ XELOX could obviously relieve leukopenia than only received XELOX regimen. Furthermore, Disodium cantharidinate and vitamin B6+ XELOX, Shenqifuzheng+ XELOX, Kangai+ XELOX, Lentinan + XELOX could obviously relieve the nausea and vomiting than receiving the XELOX regimen alone. However, CHIs combined with XELOX regimen could not experience the better clinical effectiveness rate than receiving XELOX regimen alone, without statistically significant between-group differences.

Gastric cancer is the second commonest type of cancer with high incidence and mortality.^[63]^ Owing to its low preoperative diagnosis rate, the majority of patients is highly prone to distant metastasis, approximately 80% of cases eventually develop into advanced gastric cancer and receive chemotherapy.^[64]^ XELOX regimen is one of the recommended regimens NCCN clinical practice guidelines in oncology for gastric cancer.^[65]^ Cap is an oral prodrug of fluoropyrimidine that is converted to fluorouracil in tumor tissue in a reaction that is catalyzed by the enzyme thymidine phosphorylase.^[66,67]^ However, some research showed that Cap is possible relative to cardiac toxicity and neurotoxicity.^[68]^ And it is reported that L-OHP could cause acute peripheral neuropathies.^[69]^ TCM has characterized by overall regulation, syndrome differentiation treatment, specimen, and centralizer. And its therapeutic selectivity can preferentially kill tumor cells and inhibit the amplification of cancer with nonsignificant drugs resistance.^[70,71]^ With respect to CHIs, they had advantages in relieving surgery complications, suppressing tumor progression, increasing the sensitivity of chemo- and radiotherapeutics, improving immunologic function, Huachansu injection, Shenqi fuzheng injection, and Kanglaite injection; on the other hand, CHIs might reduce toxicity and enhance efficacy in combination with radiotherapy or chemotherapy.^[17,18]^ Huachansu injection was a water-soluble preparation from toad skin that with a long tradition in China, some recently released studies reported that the major constituents in Huachansu injection, namely peptides, nucleic acids, tryptamines, and bufotalins had the antitumor activity. Also, bufadienolides showed significant inhibition rates on the growth gastric tumor growth in vivo.^[72,73]^ The molecular mechanism of Huachansu injection might be associated with inhibiting the proliferation of BGC-823 and inducing the expression of miR-494, remarkably, miR-494 possibly is a potential molecular target for cinobufacin against gastric cancer.^[74]^ Correlative studies have reported that Kangai injection was made from *ginseng*, *Astragalus*, *Sophora flavescens*, and its active components mainly include Astragalus saponins, ginsenoside, and matrine. And Kangai injection had influence on the enzyme activities of macrophages and morphology in rats’ spleen and thymus.^[75,76]^ As for Compound Kushen injection, it was composed of *Rhizoma Heterosmilacis Japonicae* and *Sophora flavescens*, exhibiting various pharmacological activities, such as antiinflammatory, antiallergic, antiviral, and antifibrotic effects.^[77]^ Moreover, previous meta-analyses confirmed that Aidi injection combined with chemotherapy could significantly improve the clinical effect of chemotherapy, reducing the incidence of adverse events.^[78,79]^ The main constituent of Lentinan injection is (1–3)-beta-d-glucan, which is a purified polysaccharide isolated from Lentinus edodes. Lentinan injection is not only a potent anticancer drug licensed in China for antitumor therapy since 1995, but also clinically administered to patients with unresectable advanced gastric cancer in Japan.^[80]^ The biological functions of lentinan include antiinflammatory activity, cellular immunity promotion, immune stimulation, and anticancer effects.^[81]^

The advantages of this study were shown in the following aspects: firstly, this is the first network meta-analysis to compare the efficacy and safety of CHIs combined with XELOX regimen for gastric cancer. Literature searches were conducted about 22 types of CHIs which have been used for cancer treatment at the present, and the inclusion and exclusion criteria were established strictly. Secondly, the retrieval of this study was relatively comprehensive. On the one hand, apart from searching the database of domestic and foreign, we also search RCTs at related academic organization websites. On the other hand, the searching words and searching strategy were amended and confirmed by expert on data retrieval methods. Thirdly, the common interventions were chemotherapy of included RCTs; the criterion of therapeutical effect met the WHO for solid tumors. Finally, this study not only analyzed the clinical effectiveness rate and the improvement of performance status, but also focused on the ADRs.

## 5 Limitation

There are certain limitations to the present NMA. First, survival time was an important end-point outcome for evaluating the curative effect against cancer; however, only 4 trails among included RCTs reported the information of survival time or follow-up. Second, this study was limited by the quantity and quality of the included RCTs, and clinical diversity still remains among included trials. And there is lack of large sample-size trails and head-to-head trails that focus on different CHIs. Third, all of the included RCTs were performed in Asian descent; therefore, it is unclear whether the conclusions of our study applicable for other populations. Despite the above limitations, our study is the first NMA that provides a complete evaluation of the clinical effect, performance status, and ADRs of different CHIs for gastric cancer patients. Nonetheless, more large-sample, multicenter and head-to-head RCTs or further mechanism study are warranted for elucidating our conclusions.

## 6 Conclusion

In summary, the current evidence shows that CHIs combined with XELOX regimen could provide treatment benefits for patients with gastric cancer. Among 13 types of CHIs, Javanica oil emulsion and Compound Kushen injection seem to be the optimal in improving the clinical effectiveness rate and performance status, and Lentinan injection is more favorable in relieving ADRs.

## 7 Author contributions

**Conceptualization:** Dan Zhang, Jiarui Wu.

**Performance of the experiments data curation:** Dan Zhang, Shi Liu, Kaihuan Wang, Xiaojiao Duan, Jiarui Wu.

**Formal analysis:** Dan Zhang, Jiarui Wu.

**Investigation:** Dan Zhang, Shi Liu, Jiarui Wu, Kaihuan Wang, Xiaojiao Duan, Bing Zhang.

**Methodology:** Dan Zhang, Shi Liu, Kaihuan Wang, Xiaojiao Duan, Jiarui Wu.

**Project administration:** Jiarui Wu.

**Software:** Dan Zhang, Shi Liu, Kaihuan Wang, Xiaojiao Duan, Jiarui Wu.

**Writing – original draft:** Dan Zhang, Jiarui Wu.

**Writing – review and editing:** Jiarui Wu, Bing Zhang.
